# Effects of single dose GnRH agonist as luteal support on pregnancy outcome in frozen-thawed embryo transfer cycles: an RCT

**Published:** 2015-08

**Authors:** Robab Davar, Maryam Farid Mojtahedi, Sepideh Miraj

**Affiliations:** *Reasearch and Clinical Center for Infertility, Shahid Sadoughi University of Medical Sciences, Yazd, Iran.*

**Keywords:** *Frozen*, *Embryo transfer*, *GnRH agonist*, *Luteal phase*

## Abstract

**Background:**

There is no doubt that luteal phase support is essential to enhance the reproductive outcome in IVF cycles. In addition to progesterone and human chorionic gonadotropin, several studies have described GnRH agonists as luteal phase support to improve implantation rate, pregnancy rate and live birth rate, whereas other studies showed dissimilar conclusions. All of these studies have been done in fresh IVF cycles.

**Objective:**

To determine whether an additional GnRH agonist administered at the time of implantation for luteal phase support in frozen-thawed embryo transfer (FET) improves the embryo developmental potential.

**Materials and Methods:**

This is a prospective controlled trial study in 200 FET cycles, patients were randomized on the day of embryo transfer into group 1 (n=100) to whom a single dose of GnRH agonist (0.1 mg triptorelin) was administered three days after transfer and group 2 (n=100), who did not receive agonist. Both groups received daily vaginal progesterone suppositories plus estradiol valerate 6 mg daily. Primary outcome measure was clinical pregnancy rate. Secondary outcome measures were implantation rate, chemical, ongoing pregnancy rate and abortion rate.

**Results:**

A total of 200 FET cycles were analyzed. Demographic data and embryo quality were comparable between two groups. No statistically significant difference in clinical and ongoing pregnancy rates was observed between the two groups (26% versus 21%, p=0.40 and 21% versus 17%, p=0.37, respectively).

**Conclusion:**

Administration of a subcutaneous GnRH agonist at the time of implantation does not increase clinical or ongoing pregnancy.

## Introduction

The number of frozen-thawed embryo transfer (FET) cycles has already been increased in modern assisted reproduction treatment (ART), which partly reflects the increased use of single-embryo transfer in a fresh in vitro fertilization (IVF) cycles, and also as a result of improved laboratory techniques. Luteal phase defect is a current problem either in ART cycles due to very low Luteinizing hormone (LH) concentrations during the luteal phase in agonist and antagonist cycles and in artificial cycle FET (AC-FET), due to the lack of corpus luteum, thus support of corpus luteum function is mandatory (1-4).

Progesterone and human chorionic gonadotropin have been used as popular and effective luteal phase support (LPS) agents. In addition, estradiol and gonadotrophin-releasing hormone agonist (GnRH-a) are adjunct products offered as LPS to improve the outcome of assisted reproduction techniques (5-7).

The exact underlying mechanism of GnRH agonist is still not clear, although it has been hypothesized that GnRH agonist either supports the corpus luteum function by inducing LH secretion by the pituitary gonadotroph cells or stimulates the endometrium GnRH receptors (7, 8). The beneficial effect of GnRH agonist in the luteal phase in IVF/Intra-cytoplasmic sperm injection (ICSI) cycles on the outcome of pregnancy has also been shown in the recipients of donated oocytes (5). Currently, available data regarding the benefit of administration of GnRH agonist on pregnancy outcome in fresh ICSI cycles exist; However this is the first study regarding the frozen-thawed embryo transfer cycles (9). The aim of this study is to investigate the beneficial effect of GnRH agonist as LPS on pregnancy outcome in FET cycles.

## Materials and methods

**Study design**

This study is a single-blind randomized clinical trial conducted at the Research and Clinical Center for Infertility, Yazd, Iran, between April 2014 to January 2015.

Tesarik, Hazout *et al*. showed that in egg-donation cycles, GnRH administration to recipients during the luteal phase (on day 6 after ICSI) increases the implantation rate from 25.1% to 36.9% and pregnancy rate from 54.3% to 67.4%. On the basis of this published data, the power was set at 80% and it was found that 100 cycles were needed in each group to detect this difference.

**Study population**

A total of 221 patients were assessed for eligibility. Exclusion criteria was patients below the age of 18 and over 40 years, oocyte recipients, patients who had systemic or endocrine disorders such as diabetes mellitus and thyroid disorders, endometriosis, submucous fibroids or intrauterine adhesions with or without history of previous surgery. Signed consent was obtained from all participants who were enrolled for randomization. This study was approved by the Ethical Committee of the Research and Clinical Center for Infertility, Yazd Iran. A computer-generated randomization table was created for the study population. Allocation concealment was ensured by the use of the day of embryo transfer.

**Figure 1:**
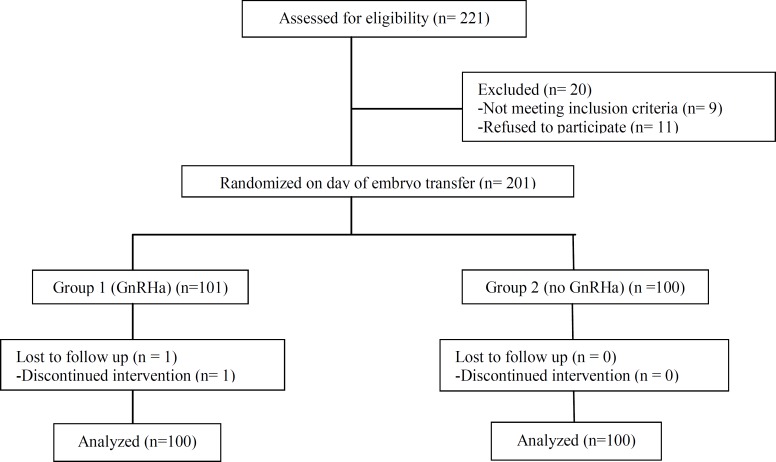
Flowchart of the study design

Patients were administered estradiol valerate 6 milligrams daily and ASA 80 mg from day 2 of the cycle, followed by daily vaginal progesterone suppositories (total dose 800 mg) since the endometrial thickness reached 8 mm. Randomization was done: group 1 (n=100) patients received 0.1 mg subcutaneous GnRHa injection (Decapepty l; Ferring, Germany) three days after embryo transfer; in group 2 (n=100), patients did not receive GnRHa. Both groups received daily progesterone suppositories (total dose 800mg), estradiol valerate 6 mg and ASA 80mg. Chemical pregnancy was diagnosed by serum β-subunit of hCG gonadotropin (β-hCG) concentration 12-14 days after embryo transfer. Clinical pregnancy was diagnosed by ultrasound detection of fetal heart activity 2 weeks after positive hCG test.

The primary outcome measure was clinical pregnancy rate and the secondary outcome measures were implantation rate, chemical pregnancy, ongoing pregnancy, and multiple pregnancy and abortion rates.

**Statistical analysis**

SPSS software (Statistical Package for the Social Sciences version 15.0, SPSS Inc., Chicago, IL, USA) was used for all statistical calculations. Chi-squared test and exact test was used for comparing categorical data. P<0.05 was considered statistically significant.

## Results

A total of 200 patients underwent FET cycles. No significant difference was found in age, BMI, cause and duration of infertility, endometrial thickness at progesterone initiation day, number of transferred embryos and embryo score (Table I).

No significant difference was found in clinical and ongoing pregnancy rates and abortion rate between the two groups (Table II).

Although clinical pregnancy and ongoing pregnancy rates were slightly higher in the GnRHa group compared with the controls, the observed differences were short of reaching statistical significance.

**Table I:** Patient demographics and cycle characteristics (mean ± SD)

	**Group 1 (GnRH agonist) (n=100)**	**Group 2 (non GnRH agonist) (n=100)**	**p-value**
BMI	23.70 ±2.85	23.76± 2.78	0.89
Age (years)	30.03±5.16	30.08±5.21	0.94
Duration of infertility (years)	8.14±4.46	7.76±4.33	0.54
Endometrial thickness at progesterone initiation day	8.24±0.38	8.30±0.46	0.33
Mean number of transferred embryos	2.38±0.62	2.45±0.63	0.43
Embryo score	16.77±1.07	16.55±1.53	0.24
Good quality embryos (%)	64	58	0.21

Qui-square test

**Table II:** Pregnancy outcome analysis in GnRHa and non-GnRHa groups

	**Group 1 (GnRH agonist) (n=100)**	**Group 2 (non GnRH agonist) (n=100)**	**p-value**
Implantation rate	14.66	13.07	0.31
Chemical pregnancy rate	27	27	1.00
Clinical pregnancy rate	26	21	0.40
Ongoing pregnancy rate	22	17	0.37
Abortion rate	5	8	0.39

* Qui-square test

## Discussion

Several studies have described GnRH agonists as LPS to improve implantation rate, pregnancy rate and live birth rate. This study assessed the effect of a single dose of a GnRHa (0.1 mgtriptoreline) on implantation, clinical and ongoing pregnancy rates in FET cycles. No statistically significant differences in the clinical or ongoing pregnancy rates were found between two groups. 

Several studies have reached similar conclusions to our study, (10). In a large randomized, study by Ata *et al*., (2008) one dose of GnRHa was injected 6 days after embryo transfer. No difference was found in pregnancy rate by GnRHa administration.

In another randomized study in IVF cycles with the long GnRHa protocol, three additional injections of 0.1 mg GnRHa on day 6 after embryo transfer did not affect the pregnancy rate (11). 

They concluded that the continuous administration of the GnRHa in the luteal phase can lead to sustained down-regulated state of the GnRH receptors in the reproductive organs, which may cause ineffectiveness of GnRHa in improving the pregnancy rate, (12) studied the effect of continuing of GnRHa in the luteal phase on the outcome of assisted reproduction technique cycles, and found that duration did not affect the pregnancy rate. In a prospective randomized study, (Aboulghar, Marie *et al*. 2015) did not confirm the value of continuous administration of GnRHa for the whole luteal phase compared to much shorter durations in other studies (13).

Several studies have reported a positive effect of administration of GnRHa in the luteal phase. In a prospective randomized study, (Tesarik, Hazout *et al*. 2006) found a higher implantation rate and live birth rate after a single dose of 0.1 mg GnRHa administered 3 days after embryo transfer in both agonist and antagonist protocols. 

The pregnancy rate, however, was not significantly improved (8). Previously, these investigators found that the administration of a single dose of GnRHa in the luteal phase increased pregnancy, implantation and live birth rates in recipients of donated oocytes in whom ovulation was suppressed and thus the corpus luteum was absent (5). 

This study was more similar to ours, since preparation of egg-donated recipients resembles the frozen thawed embryo cycles. The hypothesis that a short-acting GnRH agonist administered to oocyte recipients as a single injection 6 days after ICSI will improve pregnancy outcomes excludes any possible effect on oocyte quality; thus the mechanism of the beneficial effect of luteal phase GnRH agonist administration might be explained by a direct effect on the embryo and/or on the endometrium (11, 14). Although molecular studies have suggested a direct effect of GnRHa on endometrial receptivity, current research failed to show any clinical relevant effect (15, 16). GnRH receptors are expressed at the mRNA level in vitro in cultured mouse embryos during the preimplantation period (morula to hatching blastocyst stages). In addition, it has been suggested that GnRH might play an important role on hCG synthesis and secretion either at placenta and preimplantation embryos. This is due to the fact that GnRH receptors are located not only in the trophectoderm, but also in the inner cell mass of the mouse blastocyst, (14).

There are two systematic reviews demonstrating that administration of a luteal phase single-dose GnRH agonist can significantly enhance IVF outcomes. The meta-analaysis, conducted by Kyrou, Kolibianakis *et al.* showed a positive effect of GnRHa on improving clinical pregnancy rate and live birth rate in both agonist and antagonist protocols. Kung, Chen *et al*. Confirmed in their study that a subgroup of patients with basal FSH >8 mIU/mL or mature oocytes ≤3 would benefit from luteal phase single-dose decapeptyl administration (9, 17). Yildiz, Sukur *et al*. (2014) compared single and sequential doses of leuprolide acetate 1 mg s.c. injections 3 and 6 days after ICSI-ET to control group following controlled ovarian stimulation (COS) with long luteal GnRH agonist protocol and resulted in higher implantation, clinical, ongoing and multiple pregnancy rates in both agonist groups (18). All available data are in fresh IVF cycles; however, this is the first prospective study designed to test this hypothesis in FET cycles which would assess the direct effect of GnRHa as the regulator of embryo-endometrial interactions and embryonic development.

Our study did not confirm the favorable effect of administration of a single dose of GnRHa in the luteal phase suggested by earlier studies (5, 19-20). In the study by Tesarik *et al* (2004) the mid-luteal GnRH agonist administration increased the implantation rate in an oocyte donation program in which the success rates were high even without this additional treatment. Razieh *et al,* (2009) did not find the possible mechanism of a single dose of GnRHa as LPS, meanwhile they believed that improvement in pregnancy rate was not related to the effect of GnRHa on the corpus luteum (20).

Larger studies are required to establish the role of GnRHa in the luteal phase especially in cases of repeated implantation failure or early pregnancy loss.
